# Not Everything that Shines is Calcium

**DOI:** 10.5935/abc.20180084

**Published:** 2018-05

**Authors:** Ilan Gottlieb, Fernanda Erthal

**Affiliations:** 1 Fonte Imagem Medicina Diagnóstica, Rio de Janeiro, RJ - Brazil; 2 Departamento de Radiologia, Casa de Saúde São José, Rio de Janeiro, RJ - Brazil

**Keywords:** Atherosclerosis / physiopathology, Vascular Calcification, Coronary Artery Disease, Heredity, Diabetes Mellitus

Coronary calcification is involved in the pathophysiological process of atherosclerosis, particularly at later stages of the disease, as part of its healing process.^1^ In fact, calcification is not required for plaque development or progression, or luminal obstruction, or even for the development of cardiovascular events - these processes, in addition to plaque instability is commonly seen.^2^ Our group showed, in a randomized clinical trial on symptomatic patients, that 20% of vessels with complete obstructions in invasive angiography had no calcification according to calcium scoree,^3^ corroborating histopathological studies that demonstrated the absence of calcification in a considerable number of the coronary plaques evaluated.^4^ Understanding or the pathophysiology of coronary disease is essential to contextualize calcium score in population studies.

These studies, however, have shown some controversial results on the rates of cardiovascular events and obstructive lesions. The reason for this apparent divergence of results is the study population. Asymptomatic patients with zero calcium score are different from symptomatic ones without calcification, who in turn, are different from patients with history of early coronary disease, diabetics or smokers with zero calcium score.

We may cite the contrasting example of two hypothetical populations - one of young adults (30-35 years old), with symptoms of precordial pain, and another one composed of elderly smokers (70-75 years old), with no cardiovascular symptom. It will be no surprise to find an overwhelmingly higher frequency of significant coronary obstruction in the group of asymptomatic elderly subjects with zero calcium score than in the first group with some coronary calcification. Also, it will be no wonder to find a higher rate of cardiovascular events in the group of 70-75-year-old subjects than in the group of 30-35-year old subjects. Such paradox is obviously grounded in the difference of the study populations - the prevalence of atherosclerosis is so much higher in the elderly than in the young group that even comparisons between subgroups with and without calcification and with and without symptoms will always reveal a higher prevalence of cardiovascular diseases and events in the group of elderly smokers than in the other one.

The attempt of using the zero calcium score as a gatekeeper for coronary computed tomography angiography (or other tests) in symptomatic patients should be considered with caution, be it in an emergency room or in an outpatient clinic. Subtle differences in population profiles, including ethnical differences, may result in considerable differences in the diagnostic and prognostic performance of a zero calcium score.^5^^,^^6^ Since calcification is only an indirect marker of coronary obstruction, differing from a direct visualization of the obstructive plaque by coronary computed tomography angiography, characteristics of the population studied become critical. An analogy can be made between the low prevalence of obstructive coronary disease in patients with zero calcium score and young women; both are mere population filters. No one would today underestimate symptoms in young women only because of a low pre-test probability.

In the current issue of the ABC, Gabriel et al.^7^ elegantly show a high prevalence of coronary disease, detected by computed tomography coronary angiography, in patients with zero calcium score. Interestingly, alcohol consumption and obesity, but not age showed an association with the presence of plaques in the absence of calcification. This has important implications on the development of prevention strategies for cardiovascular diseases.

Finally, the use of calcium score for detection of coronary calcification is an important tool for stratification of cardiovascular risk in asymptomatic individuals, especially because the score is an easy and low-cost instrument. However, in the context of atherosclerosis, disease status goes beyond calcification and, definitely, not everything that shines is calcium.
